# Measurements of Deposition, Lung Surface Area and Lung Fluid for Simulation of Inhaled Compounds

**DOI:** 10.3389/fphar.2016.00181

**Published:** 2016-06-24

**Authors:** Eleonore Fröhlich, Annalisa Mercuri, Shengqian Wu, Sharareh Salar-Behzadi

**Affiliations:** ^1^Center for Medical Research, Medical University of GrazGraz, Austria; ^2^Research Center Pharmaceutical Engineering GmbHGraz, Austria

**Keywords:** *in silico* modeling, inhalation, lung surface area, deposition, lung lining fluid

## Abstract

Modern strategies in drug development employ *in silico* techniques in the design of compounds as well as estimations of pharmacokinetics, pharmacodynamics and toxicity parameters. The quality of the results depends on software algorithm, data library and input data. Compared to simulations of absorption, distribution, metabolism, excretion, and toxicity of oral drug compounds, relatively few studies report predictions of pharmacokinetics and pharmacodynamics of inhaled substances. For calculation of the drug concentration at the absorption site, the pulmonary epithelium, physiological parameters such as lung surface and distribution volume (lung lining fluid) have to be known. These parameters can only be determined by invasive techniques and by postmortem studies. Very different values have been reported in the literature. This review addresses the state of software programs for simulation of orally inhaled substances and focuses on problems in the determination of particle deposition, lung surface and of lung lining fluid. The different surface areas for deposition and for drug absorption are difficult to include directly into the simulations. As drug levels are influenced by multiple parameters the role of single parameters in the simulations cannot be identified easily.

## Introduction

Drug delivery by non-invasive alternative routes, such as dermal, oral and pulmonary delivery has much improved in the last years. Compared to the invasive routes, intravenous injection, intramuscular, subcutaneous application, etc. alternative routes have a greater patient compliance because they do not need attendance at the doctor's office and they are less painful than parenteral applications. Drug delivery by non-invasive routes has been improved due to the development of formulations with specific profiles (immediate release and modified release), co-administration with inhibitors, absorption enhancers and new devices for application (inhalers, needles). Furthermore, *in silico* methods have been developed in the last decades, which allow designing specific molecules, and prediction of absorption, tissue distribution, metabolism, excretion and toxicity to a reasonably good degree. Simulation programs, such as GastroPlus™, SimCYP®, PK-SIM®, Matlab®, Stella® and ChloePK® can simulate physiologically based pharmacokinetics (PBPK) of drugs applied mainly by the oral route, based on a mixture of *in silico, in vitro* and *in vivo* data as input parameters (van de Waterbeemd and Gifford, 2003; Kostewicz et al., 2014). For example, *in vitro* measured and/or *in silico* predicted physico-chemical parameters like logP and solubility for the compound and *in vivo* pharmacokinetic parameters for the exposed individual are combined in a single modeling. In general, the extent of inter-individual differences can be included in the simulation by modification of physiological parameters such as: tissue volumes and composition; physiological flow rates, tissue:blood partition coefficients, enzymes and transporters expression levels and filtration rates (Lipscomb et al., 2012; Reddy et al., 2013). The mechanistic PBPK models provide a physiological framework, which facilitates the incorporation of all the relevant Absorption, Distribution, Metabolization, and Elimination (ADME) processes, when the respective data are available (Jones et al., 2009; Kostewicz et al., 2014).

Compared to oral application, prediction of plasma profiles of inhaled drugs is rarely reported. However, several software have been developed to calculate these values, including computational fluid dynamics (CFD), GastroPlus™, and other compartmental pharmacokinetics/pharmacodynamics (PK/PD) models to calculate these values (Patterson, 2015). These models use airway thickness, surface area, transporter activities, lysosomal degradation, and mitochondrial activities as physiological parameters (Yu and Rosania, 2010). Several biological parameters like the permeation of the epithelial barrier can be calculated by software programs or determined experimentally using either cell monolayer or tissue explants (Fröhlich et al., 2012) and physiologically relevant exposure conditions for pulmonary exposure can be developed from existing set-ups (Fröhlich and Salar-Behzadi, 2014). In addition to absorption area and fluid available for dissolution, distribution and deposition of inhaled particles in the respiratory system determines drug concentration at the pulmonary barrier. Measurement of particle deposition *in vivo* is technically complicated but software solutions are available to help in the prediction of lung deposition. There are, however, no alternatives to *in vivo* determinations of lung surface area and lung lining fluid. This review will discuss the experimental techniques and required data for the determination of lung surface area and lung lining fluid as well as the modeling of particle deposition in the lung. The impact of critical parameters on the estimations and developed models will be also reviewed.

## Particle deposition in the lung

Several *in vivo* methods can determine particle deposition in the lung based on the use of radioactively labeled aerosols. The methodology is technically demanding, needs specific tracers and is expensive. Furthermore, available techniques such as single-photon emission computed tomography (SPECT), positron emission tomography (PET), and γ-scintigraphy provide different information. Data are mostly indicated as total lung deposition, comprising deposition in the conducting and the peripheral airways. The penetration index (PI; the ratio between deposition in conducting and peripheral airways) provides information to which extent the particles reach the alveoli, where absorption mainly takes place. The available technologies have different advantages and limitations; planar (two dimensional) γ-scintigraphy is the least expensive technique, but does not allow good separation between peripheral and medium/small airways because these regions overlap in planar view (Hickey and Swift, 2011). However, 3D information in γ-scintigraphy can be obtained by taking anterior-posterior and lateral images (Phipps et al., 1989). SPECT and PET are 3-dimensional techniques and, therefore, provide better spatial information. The main disadvantage of SPECT is the long imaging time (~20 min compared to 5 min with the γ-camera). During this time, the particles in the upper airways can be cleared (Ruzer and Harley, 2013). PET is particularly complex because cyclotron for radioactive labeling of imaging agent with short half-life, which should be used immediately after synthesis, is not available at many hospitals. Differences in the determination methods were analyzed in more detail by Biddiscombe et al. (2011). The authors pointed out that different approaches result in different deposition values and that methodologies, therefore, should be standardized to facilitate data comparison between laboratories and normalize data. It is for instance known that 3D imaging identified greater differences in the PI between small (Mass Median Aerodynamic Diameter (MMAD) of 2.6 μm) and large (MMAD = 5.5 μm) particles, than 2D imaging.

Due to the fact that *in vivo* lung deposition measurements are cost-intense, need time for evaluation and are not well standardized, there is an arising interest for computational models. The majority of such models can be divided into empirical, mechanistic, and stochastic ones. Empirical models are based on mathematical equations fitted to experimental data. Using these models, pathways in the respiratory tract are considered as identical with linear dimensions, and the deposition is calculated in the whole lung. The particle deposition in individual airways is calculated by using analytical deposition equations for pre-specified flow conditions and the average behavior of particles are calculated. Realistic description of lung structure and physiology is used for the development of mechanistic models, taking into account different breathing scenarios and parameters. Moreover, fluid and particle dynamics are correlated with respiration by simplified expressions for calculation of resulting particle motion (Rosati et al., 2004). These models are based on either idealized descriptions of lung morphology and physiology by CFD, or calculation of inhaled aerosol flow by considering the fate of a population of particles or an individual particle (Eulerian and Lagrangian models, respectively). In the models branching angles are included, which are difficult to determine in small airways and change from resting to deep inhalation. The angle of the tracheal bifurcation decreases from a value of 70° up to 10° upon deep inspiration (Breatnach et al., 1984; Holbert and Strollo, 1995). Both Eulerian and Lagrangian models can be used for modeling the whole lung or a local scale approach (Martonen et al., 1997; Zhang and Kleinstreuer, 2002; Hofmann, 2011). Examples for the Eulerian approach for the simulation of deposition at the whole lung level are the deterministic generation-based models (Yeh and Schum, 1980; Hofmann et al., 1989; Martonen, 1993), and the one-dimensional trumpet model (Egan and Nixon, 1985; Mitsakou et al., 2005). In all these cases the intersubject variability of physiological and morphological data is a limiting factor. The description of lung pathway can be also used for deterministic or stochastic models (Bradley et al., 1981; Hofmann and Koblinger, 1990, 1992; Koblinger and Hofmann, 1990; Asgharian et al., 2001; Longest et al., 2004; Mitsakou et al., 2005; Zhang et al., 2008, 2009; Hofmann, 2011; Longest and Holbrook, 2012). Stochastic modeling is a great step toward the improvement of the problem of intersubject variability, by using random variation of the geometry of the airways for incorporating the biological variability. Different deposition models and the required physiological and morphological data with the evaluation of their advantages and drawbacks have been extensively reviewed elsewhere (Rosati et al., 2004; Hofmann, 2011) and therefore are not the focus of this review. The frequently used deposition models for the development of software programs are single path and multiple path models. Single path models are based on empirical and semi-empirical correlations. An important example for single path models is the development of a whole lung model belongs to International Commission on Radiological Protection (ICRP) for deposition and retention of inhaled radioactive particles (ICRP, 1994). Calculation is based on a five compartment model, comprising the anterior nose; the posterior nasal passages together with larynx and pharynx; the bronchial regions; the bronchioles and the alveolar region. An alternate model has been developed by the National Council on Radiation Protection and Measurements (NCRP) (National Council on Radiation Protection, 1997). One of the main differences between these models is that the NCRP model divides the lungs down to several airway generations and, thereby, includes considerably more detail of the lung geometry than the ICRP model. The ICRP model classifies the empirical correlations into two subcategories: those governed by the aerodynamic diameter (for larger aerosols that are impaction- or interception-driven) and those governed by the thermodynamic diameter (for smaller aerosols driven by thermodynamic diffusion). Both models take also into account the amount of clearance through different regions of the lungs. Improvements of the ICRP model regarding shape factor, size distribution and correlation of lung parameters to height have been included in a more recently published model (Guha et al., 2014). In this model a correlation was developed between height and age, allowing calculation of lung deposition in all subjects as a function of height rather than age. Flow rate and tidal volume were functions of height and activity (sitting, sleeping, and light exercise). The ICRP model is considered as a standard model for routine inhalation dosimetry assessments (ICRP, 1994) and has been also integrated in software programs for calculating the deposition rate of pharmaceutical aerosols. As input data the ICRP model uses physiological parameters and environmental factors. Physiological parameters, which can be also varied by the user are age, tidal volume, airflow, and activity, also considering the both nose- and mouth-breathing. The environmental factors are aerosol size and shape. The ICRP software uses 78 m^2^ as lung surface area (Guha et al., 2014).

Multi-path models have been developed to provide a more realistic lung-modeling than the single-path approach. In multi-path modeling, the lung asymmetry branching pattern and path variation have been taken into account. This results in more realistic determination of average deposition fractions. However, the validation of such models with *in vivo* data is only possible for total or regional deposition, but not at airway generation level. An example for such models is the Multiple Path Particle Dosimetry model (MPPD) (Asgharian et al., 2001; Price et al., 2002). The parameters used by MPPD to calculate deposition comprise four areas: the type of airway morphometry, the particle properties, the exposure, and the possibility to evaluate the deposition or the deposition and clearance of the particles. Regarding the airway morphometry, there is the possibility to select between the human and the rat species. More in the specific, five different morphometry are available for the human and only one for the rat.

Morphometry includes the number of airways, individual airway dimensions, spatial structure of branching network and ventilatory conditions. Considering the selected morphometry and resulting specific air flow patterns (laminar or turbulent), it is possible to choose between a uniform and a non-uniform air flow velocity. Other morphometric parameters that can be varied are the FRC (Functional Residual Capacity) and the URT (Upper Respiratory Tract) volume. The physico-chemical characteristics of the particles, which are used to determine their lung deposition in MPPD, are the density, the particle mean diameter (either as Count Mean Diameter (CMD), Mass Mean Diameter (MMD) or MMAD) and the Geometric Standard Deviation (GSD). Inhalability of nanoparticles can also be taken into account. The type of exposure can be selected between constant and variable, thus to simulate, respectively, the deposition from breathing at a fixed tidal volume and breathing frequency, or from an environmental exposure which can change over time. For the simulation of the deposition under constant conditions, the parameters that can be defined are: the body orientation in the space, the aerosol concentration, the breathing frequency, the tidal volume, the inspiratory and pause fractions and the breathing scenario. For the variable exposure, the parameters to be defined are: the time of exposure, the particle concentration, the breathing frequency, the tidal volume, the inspiratory and pause fractions, the breathing scenario and the time indication (activity or hourly pattern). Finally, the user can select to investigate only the deposition or to account also for clearance of the particles. The latter case can be selected only in the case of constant exposure. The parameters that have to be defined comprise the mucus velocity at the trachea, the type of clearance and settings for the exposure time. The greater flexibility and most importantly the possibility to include breath holding time makes the MPPD model useful for calculation pharmaceutical aerosol deposition (e.g., Ahmed et al., 2012; Longest and Holbrook, 2012; Wu et al., 2013). A specifically adapted version of the MPPD model can also take hygroscopic growth, coagulation and evaporation of semivolatiles into account (Kane et al., 2010).

Not only deposition, which depends on particle properties and physiological parameters, but also other parameters of the lung linked to absorption, namely internal lung surface (absorption area) and the amount of fluid that covers the pulmonary surface, are difficult to determine *in vivo*.

## Absorption area

In opposite to respiratory tract, the surface area of the gastrointestinal tract has been determined many years ago and have been corrected only recently by Helander and Fandriks (Helander and Fandriks, 2014). The group determined the surface area of stomach, small and large intestine as 35 m^2^, with a contribution of 2 m^2^ for the large intestine (Helander and Fandriks, 2014), while the older data indicated areas of small and larger intestine of 120 m^2^ and 4 m^2^, respectively (Niazi, 2007). These differences were caused by the different evaluation methods. Older techniques distended the intestine of cadavers with a pressure of 40 cm and fixed the tissue with an aqueous solution of 4% formalin (Wilson, 1967). Samples of this tissue were taken at certain intervals, embedded in paraffin and sections cut and stained for stereological histology. Values obtained by the stereological calculations were corrected for shrinkage of the tissue due to fixation. The new studies used radiological investigations, supplemented with studies of the microscopical structure for their analysis. Most importantly, parameters had not been determined in complete relaxation of the gastrointestinal tract (cadavers or surgery), but in conscious individuals.

Similar technological improvements are not available for measurements of the lung surface and stereological histology is still the preferred method to determine the internal surface of the lung. The technology has been developed in the 1960s and included the following steps: lungs were fixed by formalin steam fixation and quantitative histological analysis of sagittal slices of 1 cm thickness was performed. The volume of fresh and fixed lungs was corrected for tissue shrinkage during fixation. The surface area estimation was based on the model that similar bodies of a given volume and surface area were enclosed at random in a unit volume. This volume was transversed by a number of lines with a known total length. The total length of interior lines and the number of intercepts were included in the calculation (Dunnill, 1962). Steam vapor fixation with formalin turned out to be better than intrabronchial instillation of 4% formalin solution, because no distortion of the alveolar spaces occurred (Hasleton, 1972). Under these conditions lung surface was determined as 24–69 m^2^. Thurlbeck indicated the internal surface of the lung as 63 m^2^ when using inflation of the lungs with 10% formalin vapor and maintenance of transbronchial pressure at 25 cm for 18 h (Thurlbeck, 1967). By combination of stereological histology with electron microscopy, Weibel indicated the internal lung surface area to be about 130 m^2^ (Weibel, 2009) in one study and 150–180 m^2^ in another (Weibel, 1980). This data led to the popular comparison of the lung area to a tennis court. However, other estimations give 1 m^2^/kg body weight as realistic value for lung surface of mammals (Lenfant, 2000). This estimation could also be valid for humans because a lung surface of 70 m^2^ has been reported (Bocci, 2011). This area corresponds to the size of a single badminton court or one half of a tennis court. To complicate the situation further, the inner lung surface markedly depends on the inhalation state. Respiratory changes in lung surface were studied by von Hayek, who reported a volume of 35 m^2^ at deep expiration and 100 m^2^ at deep inspiration (von Hayek, 1960). The strong dependence of the lung area from the respiration state could be an explanation for the variable volumes indicated in the literature. Data from freshly excised lungs using 20 cm H_2_O for full inflation of the lung show that the choice of 25 cm H_2_O transbronchial pressure for the determination of the internal lung surface should be sufficient to allow the evaluation of the entire lung (Choong et al., 2007). Lower pressures of 14.0–20.6 cm H_2_O were used to study ventilation mechanics studied in the explanted lungs. Pressures below 30 cm H_2_O are also recommended in mechanical ventilation of patients in order not to damage the respiratory system (Malhotra, 2007).

## Distribution volume and protein content

Distribution volume and binding to proteins are relevant parameter for the availability of drugs (Smith et al., 2010). The volume of fluid in the different parts of the gastrointestinal tract have been determined in post-mortem studies, using isotopes and, more recently, magnetic resonance imaging (MRI), which possess sufficient resolution to determine these values (Sutton, 2009). Catheters measurements indicated 120–350 ml of residual water in the small intestine (Dillard et al., 1965) while MRI studies reported 10–250 ml (mean 90 ml) in one study and 25–350 ml (mean 165 ml) in another (Hoad et al., 2007). Variations in these values appear to be due to the different study design and time points of the measurements and not to different determination techniques. Particularly the time between water intake and measurement was important because water is readily absorbed from the small intestine and measurements at later time points result in smaller measured volumes. In regions with less pronounced absorption like the colon, variations in the fluid content between the studies were much smaller (22–30 ml) (Wang and Urban, 2014). Gastrointestinal fluid consists of secretions from the large gastrointestinal glands (pancreas, liver), submucosal glands (Brunner's glands) and intraepithelial mucus-producing cells. There are changes in pH and ion concentrations along the gastrointestinal tract, but the average total protein content in the fasted state was relatively constant at 1.2 mg/ml with variations between 0.8 and 2.4 mg/ml (Ulleberg et al., 2011). Gastric juice contains mainly pepsin (0.8–1 mg/ml) and 1.5 mg/ml mucin (Vertzoni et al., 2005). Compared to plasma total protein (60–80 mg/ml) or albumin levels (35–50 mg/ml) in blood (Busher, 1990), the concentrations in gastrointestinal fluids can be considered as low and suggest only low drug binding.

Determination of the lining fluid in the lung (LLF), also called epithelial lining fluid (ELF) or airway surface liquid (ASL), is more complicated than for the gastrointestinal fluids because lung volumes are much smaller. The total volume of LLF can be estimated based on bronchoalveolar lavage (BAL) or by extrapolation of the thicknesses of the fluid layer covering the respiratory epithelia. BAL is a diagnostic technique that uses instillation of sterile saline (0.9%) solution commonly into the segmental bronchus of right middle lobe with the aim to characterize cells but also to determine composition of the LLF and measure drug levels. The procedure has been refined over time; older protocols used the instillation of 3–7 l of fluid with 500 ml aliquots while current protocols recommend 200–240 ml (Klech and Pohl, 1989). This adaptation was needed because concentrations of protein and drug levels are based on the concentration of urea. Urea as non-polar small molecular weight molecule crosses the alveolar epithelium and is expected to be present in the same concentration in the blood and in the lung fluid. However, urea also leaks into the airspace and in this way may reach higher levels than in the blood with the consequence of overestimation of the LLF volume (Baldwin et al., 1991). Volume and duration of the dwell time are crucial parameters for the concentration of urea in BAL, and dwell times < 30 s are recommended (Tyvold et al., 2007). The volume of the BAL is further relevant because upon instillation of 300 ml saline 100 ml of BAL contain only 1 ml of LLF (Rennard et al., 1986). Determinations of the LLF volume based on the thickness of the fluid layer on top of the epithelia require a technique that does not alter the native structure of the LLF. Traditionally, transmission electron microscopy has been used using fixation with glutaraldehyde and staining with OsO_4_ in perfluorocarbon to preserve the mucus layer. To avoid tissue shrinkage by the fixation, cryofixation instead of chemical fixation has been developed (Kesimer et al., 2013). Cryofixation determined the thickness of the periciliary layer of the bronchi with 10–11 μm while older measurements using conventional fixation indicated 7 μm (Tarran et al., 2005). Furthermore, cryofixation allowed the visualization of the substructure of this layer, while the fixation with glutaraldehyde and staining with ruthenium red could not (Kesimer et al., 2013). Most published data were obtained with conventional fixation technique. The thickness of the lining fluid layer was reported as 5–10 μm in the conducting airways and 0.01–0.08 μm in the alveoli (Olsson et al., 2011). Other data report a 10–30 μm thick layer in the trachea, 2–5 μm in the bronchi, and 0.1–0.2 μm in the alveoli (Wauthoz and Amighi, 2015), 10 μm in the upper airways, 3–5 μm in the alveolar ducts and 0.3 μm in the alveoli (National Research Council, 1977) or 8.3–6.9 μm in bronchi and 1.8 μm in bronchioles (Hoffmann et al., 2014). Patton and Byron reported 8 μm for bronchi, 3 μm for terminal bronchioles and 0.07 μm for alveoli (Patton and Byron, 2007). The indication of an average thickness for bronchi does not reflect the physiological condition of a highly variable and partly discontinuous mucus layer. Focal increases of the layer of 20 times can occur and some small bronchi may completely lack a mucus layer (Hiemstra, 2010) and could cause heterogenous absorption of the drugs.

There is currently no optimal method to determine the volume of the LLF. Calculation by the urea method poses the problem of overestimation due to leakage of urea. Estimations based on the thickness of the lining fluids needs to avoid fixation artifacts and are complicated by the variable indications of the entire lung surface area. Most existing data were obtained with the measurement of urea in BAL. In the literature different volumes of 12 ml (Schlesinger, 1992), 20–40 ml (Lenfant, 2000), 25 ml (Walters, 2002), 10–30 ml (Olsson et al., 2011) and 17–20 ml (Bocci, 2011) have been indicated. Greater variations of 15–70 ml were given by Bohr et al. (Bohr et al., 2014). Based on the body weight-dependent data obtained in sheep (0.37 ± 0.15 ml/kg), a 70 kg human would possess 26 ml of LLF (Stephens et al., 1996). This study used the instillation of the impermeant tracer ^125^I-albumin in perfused postnatal sheep lungs and changes in the tracer concentration were measured. Using low temperature scanning electron microscopy Fronius et al. calculated the LLF volume in rat lungs based on the height of the fluid layer (Fronius et al., 2012). Animal experiments may offer a platform to establish new techniques for determination of these data.

**Figure 1:**
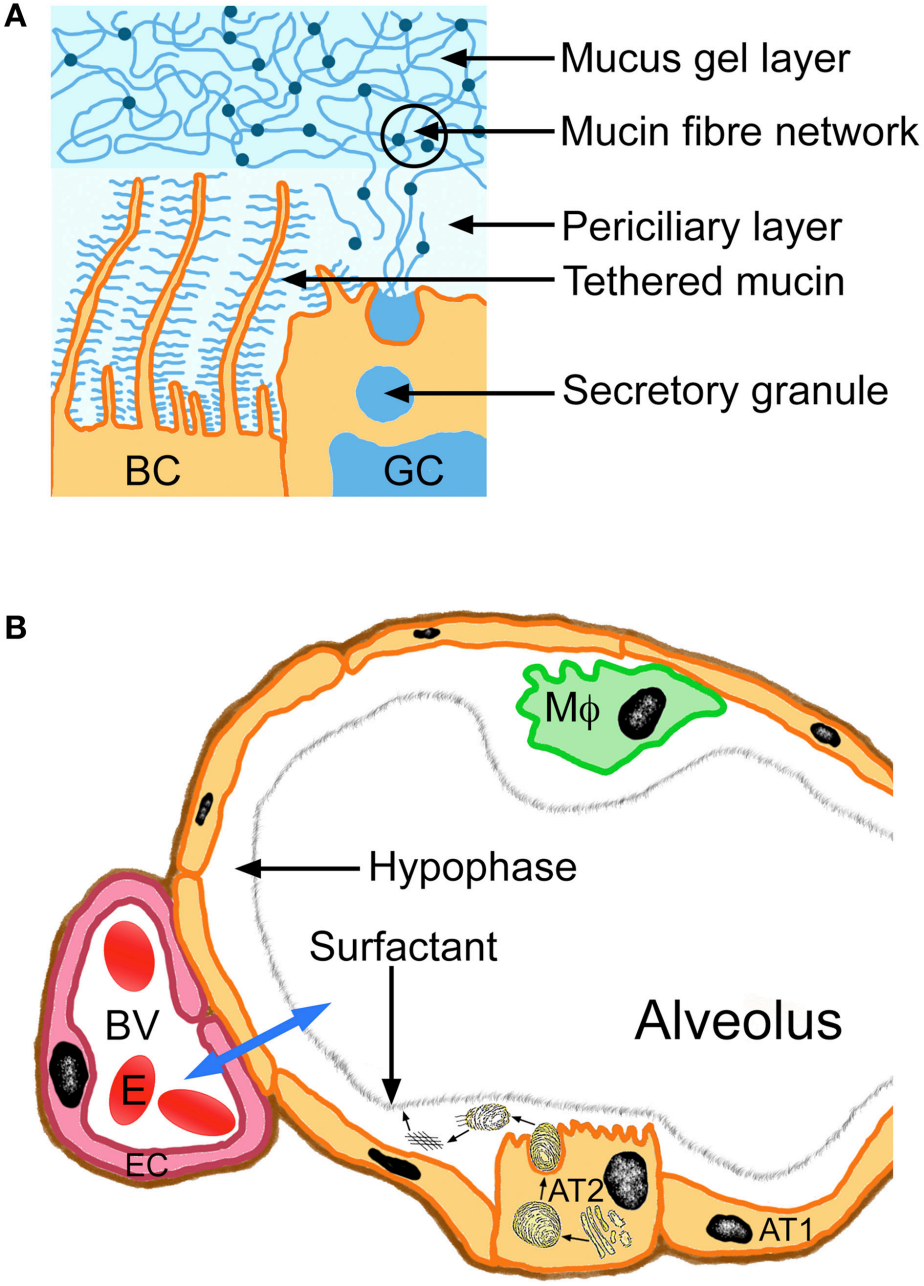
**Composition of the lung lining fluid of large airways (bronchi, A) and alveolus (B)**. The blue arrow indicates exchange between alveolus and blood vessel (BV). Alveolar type I cells (AT1) represent the predominating cell type of the epithelial lining of the alveolus. Surfactant production (small arrows) occurs in endoplasmic reticulum and Golgi apparatus of the alveolar type II cells (AT2) and the surfactant layer self assembles upon secretion from the cells. BC, bronchial epithelial cell; EC, endothelial cell; MΦ, alveolar macrophage, erythrocyte (E).

LLF has a heterogeneous composition that varies from the larger to the smaller airways. Large airways possess periciliary layer and gel mucus layer (Figure 1A). The periciliary layer consists mainly of water with antibacterial factors, ions and contains tethered mucins MUC1 and MUC4 and heparin sulfate (Rubin and Henke, 2016). The ion content of the layer is regulated by sodium uptake via the sodium channel and chloride export transporters (calcium-activated chloride channel and cystic fibrosis transmembrane ion conductance regulator). The mucus gel layer consists of 97% water and 3% solids, representing polymeric mucins MUC5AC and MUC5B (Fahy and Dickey, 2010; Button et al., 2012). The network is formed by entanglement and non-covalent calcium-dependent crosslinking of adjacent polymers. The consistency of normal mucus with 3% solids corresponding to the viscosity of egg white; in pathologies, solids in the mucus can increase up to 15%, which results in a consistency of the mucus similar to rubber gum. The LLF of the alveolar region is composed of a watery layer (hypophase) and surfactant (Fehrenbach, 2001). The hypophase contains surfactant proteins, complement proteins and antioxidants (Kobzik, 2007) and provides the milieu for alveolar macrophages that migrate on top of the alveolar epithelial cells (Fehrenbach, 2001). The correct position of the macrophages inside the hypophase was only realized when perfusion fixation instead of immersion fixation of the lung samples was used (Filippenko, 1978) and demonstrates that improved analytic methods may help to obtain more relevant physiological data. LLF consists of products of alveolar cell type II (Figure 1B) and transudation of fluid from alveolar capillaries (Hiemstra, 2010). Due to the variations in the employed BAL protocols total protein levels were given as 5.3 mg/ml and 7 mg/ml to 9.0 mg/ml in healthy adults (Holter et al., 1986; Bocci, 2011; Olsson et al., 2011). Older protocols using larger BAL volumes measured 1.3 mg/ml (Fick et al., 1984) consistent with the theory that greater instilled volumes lead to overestimation of the LLF volume and underestimation of protein concentrations. The protein content of the LLF in the alveoli (5.35 mg/ml) was higher than in the lining fluid of bronchus (3.66 mg/ml) (Olsson et al., 2011). According to the hypothesis by Baldwin et al. protein levels in the ELF represent 6.5–8% of the plasma concentration, which was quite close to the experimental values of 11.7 mg/ml and 7.9 mg/ml determined in their study (Baldwin et al., 1991). Other studies report albumin levels in ELF of 3.0 ± 1.0 mg/ml, 3.2 ± 1.7 mg/ml, and 3.7 ± 0.3 mg/ml (Rennard et al., 1986; Chastre et al., 1987; Lamer et al., 1993). These values indicate a low drug binding of ELF. Blood and LLF are in constant exchange to maintain composition of the hypophase because alveolar fluid is replaced 20 times per day due to loss by respiration (700 ml/day) (Fronius et al., 2012).

## Effects of simulation parameters on deposition and pharmacokinetic profiles

Several parameters affect the modeling of lung deposition, including the selected lung morphology, the respiratory parameters, determining air flow pattern through the lung and the clearance velocity, particle properties such as size and shape of particles and the deposition mechanisms. Mathematical calculations for deposition commonly refer to spherical particles and selected morphometric lung models for healthy, adult subjects. The major limitation for the application of deposition models is the intersubject variability of morphological and physiological parameters, which affects the validation of models with experimental data (Rosati et al., 2004; Hofmann, 2011). Moreover, linking the simulation data (deposition and pharmacokinetics) to the used lung surface area and lining fluid is problematic. In the MPPD software calculations are based on 57.22 m^2^ for human alveolar surface and 0.297 m^2^ for rats (EPA United States Environmental Protection Agency, 2004), while another group indicated 102 m^2^ and 0.4 m^2^ as default lung surface area of this software for humans and rats, respectively (Chen and Chen, 2016). Differences in lung deposition are, however, not only influenced by lung surface area and airflow but also by fluid dynamics of the inhaled air. Comparing different simulation programs Majid et al. noted prominent variations in deposition; 100% variation was due to differences in diffusion deposition and 300% of variations due to impactation (Majid et al., 2012). In addition to that, the dose at the alveolar epithelium is not only determined by deposition but also by clearance. Hofmann and Asgharian (2003) used an asymmetric, multipath model for computational assessment of mucociliary clearance velocities in bronchial airways of the human and rat lung (Hofmann and Asgharian, 2003). The experimental slow bronchial clearance values of particles smaller than 6.7 μm were explained by the delayed mucociliary clearance of particles deposited in the most peripheral conductive airways, but could not be fitted with the computational data. For acinar deposition (deposition in airways that are partly or fully alveolated), computational predictions were lower than the experimental values. This might be caused by translocation of particles initially deposited in the bronchioles to the acinar region because of the slow bronchial clearance in bronchioles (Hofmann and Sturm, 2004). The interstitial/sequestration model considers the slow clearance in bronchioles (Kuempel et al., 2006) and differs in this respect from MPPD and ICRP software. The interstitial-sequestration model has been developed as improvement of the ICRP model for insoluble materials (Gregoratto et al., 2011). According to this model 35% of the deposited material remains sequestered in the interstitium.

Differences in surface area of the human lung have practical consequences on the doses that are applied to animals in order to create realistic exposure scenarios. For this calculation the dose adaptation factor (DAF) is calculated using the ratio of minute ventilation (VE; animal/human) multiplied by the ratio of deposition fraction (DF; animal/human) and by the normalization factor (NF; area human lung/ area animal lung).

$$\begin{array}{l} \text{DAF }=\;\frac{{\left({\text{V}}_{\text{E}}\right)}_{\text{A}}}{{\left({\text{V}}_{\text{E}}\right)}_{\text{H}}}\times \frac{\text{D}{\text{F}}_{\text{A}}}{\text{D}{\text{F}}_{\text{H}}}\times \frac{\text{N}{\text{F}}_{\text{H}}}{\text{N}{\text{F}}_{\text{A}}} \end{array}$$

For calculation of the NF various combinations of human and rat surface areas have been used in the literature, e.g., 62.7 m^2^ for humans and 0.409 m^2^ for rats (Ji and Yu, 2012), 143 m^2^ for humans and 0.48 m^2^ for rats, 62.7 m^2^ for humans and 0.55 m^2^ for rats (Yu, 1996) and 79 m^2^ for human lung and 0.29 m^2^ for rat lungs (Jarabek et al., 2005). Due to the fact that lung area can be more accurately determined in rats than in humans the values used in the studies varied by a factor of 1.8, while maximum and minimum human values for humans varied by a factor of 2.5.

The dose adaptation factor, however, also contains other parameters and the effect of different lung areas used for the normalization factor might be compensated or amplified by changes in the ratios of minute ventilation and deposition fraction. The U.S. Environmental Protection Agency summarized studies on minute ventilation in rats; data obtained by plethysmography in studies between 1960 and 1978 varied between 0.05 and 0.237 L/min (Arms and Travis, 1988). Minute ventilation in humans ranged between 6.02 and 7.5 L/min. Less pronounced inter-study differences (0.14–0.39 L/min; studies 1964–1992) in rat minute ventilation were observed in a report of the Defense Research Establishment (Bide et al., 1997). Minute ventilation is usually calculated based on body weight using recommended allometric equations. Minute ventilation normalized to body weight varied relatively little between different studies, 0.64–0.8 L/min/kg for rats and 0.09–0.13 L/min/kg for humans, indicating that these differences might not have a great effect on the dose adaptation factor. The ratio of the deposition fraction is influenced in a complex manner as described above.

The link between lung lining fluid and results in pharmacokinetic studies is complicated by the fact that usually plasma levels are predicted. Lung tissue levels are not easy to obtain in humans; the disadvantages of BAL measurements have already been mentioned, lung biopsies are not representative for the entire lung and represent only one time point and lung microdialysis is a highly invasive technique (Feuerstein and Zeitlinger, 2011). Lung microdialysis would be the ideal technique because continuous measurements of lung concentrations are possible. Since the probe for microdialysis sampling of the interstitial fluid has to be inserted under visual control during thoracotomy, only very few data have been generated and drug levels are usually measured in blood samples. Blood levels are usually very low, caused by multiphasic absorption and downstream of the lung. Inhaled drugs are absorbed by branches of the pulmonary artery, which runs in parallel to the bronchial tree, and then follow the blood stream through the left atrium of the heart, the left ventricle, the aorta, and the arteries and capillaries of the upper extremity to reach the cubital vein, where blood samples are collected.

A variety of parameters determine drug plasma levels and the influence of specific parameters to the results of the prediction is difficult to discern. However, when looking only on absorption a potential link might be apparent. The study by Gaohura et al. predicted the ratio of drug concentrations in LLF to plasma and the ratio of LLF: lung tissue concentrations using a multicompartment lung model (Gaohua et al., 2015). In their study the authors predicted these ratios for tuberculostatic drugs based on 25 ml lining fluid and 140 m^2^ lung surface area. Results were in reasonable agreement (within 2.5-fold) for rifampicin, ethambutol, isoniazid, and erythromycin and too low for itraconazole, pyrazinamide, and clarithromycin (13-fold, 16-fold, and ~26-fold). When comparing simulated LLF and peak plasma concentrations of rifampicin *in vivo* plasma levels were at the maximum 2-fold higher and LLF concentrations almost 8-times higher. Lowering the pH of the lining fluid and inclusion of transporter activity in the model reduced the degree of underestimation of the LLF:plasma ratio but the trend remained and it might also be suggested that the great surface area that was used in the predictions played a role. Under the assumption that a greater lung surface enables better uptake of the compounds and result in higher plasma levels the LLF:plasma ratio would decrease. Another reason could be that the volume of LLF in the model was too low and, as a consequence, the drug concentration to high. Since the equations of the model were not indicated and other parameters (published data, blood flow rate, ventilation/perfusion, tissue volume, extrapolated permeability etc.) also influence the simulation, such a conclusion, however, remains highly speculative.

## Conclusion

Variable data for lung surface and LLF in the literature illustrate the problems in choosing the right preset values for simulation of lung absorption. Reported variations in the LLF result in differences of 7 times in the potential dissolution volume of the drug. For the absorption area differences are in the same order of magnitude (7.5 times). Improved and new techniques helped in a better determination of physiological data and new technologies may prove that the current textbook data are not correct. Data derived from animals, where more invasive methods can be applied, may be helpful to better include the dynamic changes of lung parameters during oral inhalation of drugs. Several factors influence deposition of particles and absorption of drugs and it is, therefore, difficult to link the effect of individual parameters to the result of the simulation. In general, a greater surface area appears more realistic when simulating therapeutic inhalation, while smaller surface areas are realistic for environmental exposure. Deep inhalation causes increase of lung surface area but is also accompanied by higher airflow and both parameters will influence particle deposition in a complex way. Furthermore, true alveolar surface available for gas is 20–50% smaller than the epithelial surface depending on the level of air space inflation. At full inflation of 140 m^2^, for instance, the “true” alveolar surface is only 70–100 m^2^ (Gehr et al., 1978). It appears likely that different surface area values are relevant for deposition and absorption because the lung surface area gets smaller after the inhalation maneuver has finished and this fact has to be taken into account in calculations of drug absorption. Intersubject variability of lung deposition is a major limitation for general using of such models for *in silico* predictions. Implementation of the dynamic changes of lung parameters in *in silico* models is a challenging and complicated task.

## Author contributions

All authors listed, have made substantial, direct and intellectual contribution to the work, and approved it for publication.

### Conflict of interest statement

The authors declare that the research was conducted in the absence of any commercial or financial relationships that could be construed as a potential conflict of interest.
